# Using comprehensive geriatric assessment for older adults undertaking a facility-based transition care program to evaluate functional outcomes: a feasibility study

**DOI:** 10.1186/s12877-022-03255-5

**Published:** 2022-07-19

**Authors:** Ying Git Wong, Jo-Aine Hang, Jacqueline Francis-Coad, Anne-Marie Hill

**Affiliations:** 1 Curtin School of Allied Health, Faculty of Health Sciences, Curtin University, Kent St, Bentley, WA 6102 Australia; 2 School of Allied Health, University of Western Australia, 35 Stirling Hwy, Crawley, WA 6009 Australia

**Keywords:** Continuity of care, Intermediate care, Transition care, Rehabilitation, Outcome measures, Comprehensive geriatric assessment

## Abstract

**Background:**

The study aimed to evaluate the feasibility of using a comprehensive geriatric assessment (CGA) in a residential transition care setting to measure older adults’ functional outcomes.

**Methods:**

A convenience sample of older adults (*n* = 10) and staff (*n* = 4) was recruited. The feasibility of using assessment tools that comprise a CGA to comprehensively measure function in physical, cognitive, social and emotional domains was evaluated pre- and post-rehabilitation.

**Results:**

10 older adults (mean ± SD age = 78.9 ± 9.1, *n* = 6 male) completed a CGA performed using assessments across physical, cognitive, social and emotional domains. The CGA took 55.9 ± 7.3 min to complete. Staff found CGA using the selected assessment tools to be acceptable and suitable for the transition care population. Older adults found the procedure to be timely and 60% found the assessments easy to comprehend. Participating in CGA also assisted older adults in understanding their present state of health. The older adults demonstrated improvements across all assessed domains including functional mobility (de Morton Mobility Index; baseline 41.5 ± 23.0, discharge 55.0 ± 24.0, *p* = 0.01) and quality of life (EQ-5D-5L; baseline 59.0 ± 21.7, discharge 78.0 ± 16.0, *p* < 0.01).

**Conclusions:**

Incorporating CGA to evaluate functional outcomes in transition care using a suite of assessment tools was feasible and enabled a holistic assessment.

**Supplementary Information:**

The online version contains supplementary material available at 10.1186/s12877-022-03255-5.

## Background

More than one-third of older people who are admitted to hospital experience significant functional decline with reduced ability to perform activities of daily living (ADLs), including bathing and dressing [1, 2]. Additionally, around 40% of older people experience poorer performance with more complex instrumental ADLs (IADLs) such as shopping and cleaning [2, 3]. This is due to factors including poor pre-admission function, presence of multiple comorbidities and development of iatrogenic problems resulting from the hospitalization [3, 4]. This functional decline has been found to be significantly associated with an increased risk of falls after discharge, reduced independence, hospital readmissions, unplanned admissions to residential aged care (RAC) homes and mortality post-hospital discharge [3, 5, 6]. Falls related injuries occurring in the post hospital period have been found to be a leading readmission diagnosis [7]. Transition care programs (TCP) have been introduced in countries such as United Kingdom (UK), United States of America (USA), Canada and Australia to provide short-term therapeutic care to older people to help them regain functional ability and return to independent living after hospital discharge [8–11]. The programs aim to reduce hospital readmissions after discharge and reduce the need for unplanned admissions to RAC [8, 9]. Transition care programs are delivered by multidisciplinary teams and include physical therapy, occupational therapy, social work, nursing and personal care [8–11] in a variety of settings. In Australia they are delivered in residential care settings (facility-based TCP) or in the older adult’s home through a community clinic [12]. In the UK TCP can be delivered at home, in a facility-based model in a residential care setting or in a hospital [13]. Older people access transition care when they are at risk of poor discharge outcomes from hospital [12]. Those entering facility-based TCP tend to be of more advanced age and comorbidity with lower levels of physical and cognitive function compared with those discharged for restorative care at home [12, 14]. Multi-disciplinary assessment considerations for facility-based TCP include the older person having the capacity to benefit from additional targeted rehabilitation to complete their restorative process, enabling their return home to independent living [9]. Transition care programs including those that are provided in skilled nursing facilities in the USA, intermediate care in UK and TCP in Australia have been shown to improve older peoples’ clinical outcomes by reducing readmission to hospital and addressing their health care needs at times of transition, especially after acute hospital admissions [12, 15–17]. However, problems associated with TCP including those undertaken in facility-based settings have been identified. These included re-hospitalizations, the functional decline of patients admitted [15, 18–20] hence the need for more appropriate resources, including therapy that is of sufficient intensity [16, 18, 21].

Facility-based TCP have been found to improve older peoples’ [8–10, 22] performance in self-care and ADL performance on discharge [10, 12]. However, mixed results have been observed for their effectiveness in improving other functional outcomes for older people. Over 60% of older people in the USA demonstrated slow gait speed and poor physical function (measured using the Short Physical Performance Battery) at discharge from a skilled nursing facility in the USA [23]. Older people who completed facility-based TCP in Australia have been found to make improvement in ADL but are more likely to be admitted to RAC and have increased mortality compared to age-matched populations [12]. Changes in self-care in ADL is measured in both Australian and UK facility-based care using the Modified Barthel Index (MBI) [9]. However, in Australia, the MBI is the only mandated assessment tool for older people undertaking TCP [12]. The MBI only assesses the older adult in the performance of ADLs [24] without assessing impairments such as strength and balance [25, 26], or cognitive ability and social and emotional wellbeing that comprise function. Older adults admitted for TCP have complex conditions that broadly impact their physical and psychosocial wellbeing [12, 13]. If specific impairments representing these domains of function are not assessed, then deficits in abilities such as cognition, mobility, endurance, balance and falls risk may not be identified and subsequently treated. Therefore, more comprehensive assessments may be required for health professionals to understand how the older person is functioning and provide individualized rehabilitation [27]. Recent reviews of TCP in Australia and the UK have identified that the current assessment outcome measures may only provide limited information about the functional ability of older people undergoing TCP [28, 29]. National audits have recommended that outcome measures used should provide a more holistic picture of older peoples’ health status [17, 28]. A recent systematic review and meta-analysis synthesized the evidence for the effectiveness of TCP on health-related outcomes for older adults undertaking facility-based TCP after hospitalization [29]. This review found that while TCP improved older adults’ performance of ADL, other functional outcomes such as cognitive ability, emotional status and physical impairments such as balance were infrequently evaluated, making it uncertain if facility-based transition rehabilitation effectively assisted older adults to regain functional independence [29].

It has been suggested that implementing comprehensive geriatric assessment (CGA) could help tailor rehabilitation to older peoples’ needs [17, 28, 30]. Comprehensive geriatric assessment is a multi-dimensional assessment that aims to evaluate older people in terms of their impairments, functional capacities and needs in order to be able to develop a holistic plan of care [31, 32]. Frameworks of CGA vary but most often address function broadly categorized under physical, emotional, cognitive, and social domains, including for example walking ability, presence of depression, memory loss and home care supports [30–32]. An umbrella review of CGA reported that most settings undertaking CGA included medical, psychological, social and functional assessments [31]. As part of CGA, a set of valid and standardized outcome measures are essential to help healthcare professionals design tailored rehabilitation programs aimed at improving the functional status of older people to maximize their independence and quality of life (QoL) [33]. These assessments should identify impairments and activity limitations and evaluate changes in all four domains of function as described by CGA [33]. Utilizing a suite of standardized assessment tools has not been evaluated to determine feasibility in facility-based TCP. Despite previous recommendations specifying adopting a more holistic approach to assessment, identifying predictors of functional change and evaluating how to address measurement challenges [16, 17, 23, 28]. Therefore, it is important to determine what range of assessment tools are feasible to perform as part of a CGA for older adults undertaking facility-based TCP. These assessments could be used to measure the changes older adults make when completing rehabilitation. With the increased falls risk among older adults post-hospital discharge, the implementation of CGA can also play an essential role in evaluating falls risk in older adults undertaking facility-based TCP [34].

The primary aim of the study was to evaluate whether measuring older peoples’ functional outcomes using CGA was feasible in a facility-based transition care setting. The secondary aim was to evaluate if the assessments identified the impact of undertaking a facility-based TCP on older peoples’ functional outcomes across physical, mental, social and emotional domains. This feasibility study was intended to inform a planned prospective observational cohort study that would implement CGA for older people undertaking a facility-based TCP.

## Methods

### Study design

A feasibility study using pre-post test design was conducted between November 1 – December 31, 2020. Feasibility studies focus on the question “can it work?” in determining the acceptability and suitability of an intervention for a given population [35]. This can include the selection of outcome assessment tools, methodological procedures and resources required [35].

### Participants and setting

The study was conducted at a 47-bed transition care facility located in Western Australia where older people were admitted for short-term restorative care. A convenience sample of older people were recruited. Older people and their families were informed about the study through advertisement using flyers placed throughout the facility. Researchers subsequently approached older people admitted to the facility to undertake a restorative TCP to provide more information about the study and answer questions about potential participation. Eligibility criteria were: being over 60 years of age, undertaking a restorative TCP for a minimum of 2 weeks, scoring > 23/30 on the Mini Mental State Examination (MMSE) [36], which has been shown to be a common cut-off value to categorize older people as having cognitive impairment [37, 38] and being able to provide written informed consent. Older people were excluded from the study if they did not speak English or were admitted to the facility to await admission to RAC or palliative care. Further restrictions due to the COVID-19 pandemic resulted in older people who were receiving care in isolation for infection control being excluded.

Health professional staff at the facility were also recruited to provide feedback about the feasibility of using the selected outcome measures. Recruitment utilized a short verbal presentation at a staff meeting and advertisement using flyers placed in the staff room. Inclusion criteria for staff were ability to communicate in English and employment at the facility in a clinical capacity for at least 3 months.

### Outcome measures

A suite of assessment tools was chosen to measure the physical, social, emotional and cognitive functional outcomes based on the CGA framework [33] and are summarized in Appendix 1 [10, 23, 39–41]. These standardized and validated assessment tools were: the Modified Barthel Index (MBI) [24], de Morton Mobility Index (DEMMI) [42], Timed Up and Go (TUG) [43], 10-m Walk Test (10MWT) [44], Lawton scale [45], EQ-5D-5L [46], Patient Health Questionnaire-9 (PHQ-9) [47], Geriatric Depression Scale (GDS) [48] Mini Mental State Examination (MMSE) [36], and the Montreal Cognitive Assessment (MoCA) [49]. The MoCA and MBI were already being used for assessment at the participating facility. These tools are helpful in identifying impairments in areas including strength, balance and cognition, which reduce safe functional ability and predispose to falls and inability to participate in activities of daily living.

As part of CGA [50], participants’ history of falls [51], number of prescribed medications [52] and medical conditions were also recorded by the nurse participants at the point of admission to the facility. Demographic information was also collected by these nurses, including age, use of walking aids, care support prior to hospital admission, living situation prior to hospital admission, previous functional status, and length of stay in hospital. For the purpose of this feasibility study, patient goals were not assessed but these were included for participants as part of the usual care provided at the facility.

Older people were surveyed to gain their feedback regarding the acceptability of the assessment procedure. Four open-ended prompts were used regarding i) time taken for the assessment, ii) ease of completing the assessment, iii) usefulness of the assessment in helping them understand their health status, and iv) suggestions and feedback to improve the assessment experience. Participating staff undertook a short survey consisting of four items, including two open ended questions, to ascertain if they found it easy to administer each assessment tool and if any adverse events occurred. The open prompts asked staff to evaluate whether they felt it was i) acceptable to administer the chosen assessment tools and ii) whether they were suitable for the facility setting.

### Data collection procedure

Older people were recruited during their first week of admission to the facility and their functional outcomes were measured using the suite of assessment tools. The occupational therapist administered the MMSE and MoCA, nurses administered the MBI and physiotherapists administered the other assessments (10MWT, DEMMI, TUG, EQ-5D-5L, Lawton scale, GDS and PHQ-9). Questionnaires were conducted face to face at the older adult’s bedside, while physical assessment items were conducted in their room and along the corridors of the facility. The functional outcomes were re-measured at discharge using the procedure previously described.

On completion of the suite of assessments the researcher conducted the feasibility evaluation survey one-to-one with participating older people and staff. Responses were noted verbatim by the researcher.

### Data analysis

Data were analyzed using Stata version 16.0 (StataCorp, College Station, Texas, USA). Quantitative data for the feasibility criteria of assessment completion and time taken were summarized using descriptive statistics and reported as frequencies and proportions. Responses to survey questions seeking categorical information, such as suitability and acceptability, were subjected to quantitative content analysis [53]. Data were extracted based on the number and frequency of categories identified within each document [53]. Results were summarized using counts and exemplar statements. The researchers engaged in dialogue to reach agreement on the coding and analysis.

Quantitative data for each functional outcome across the physical, social, emotional and cognitive domains were summarized using descriptive statistics. Data were not normally distributed, except for the EQ-5D-5L, therefore changes in functional outcomes between baseline and discharge were analyzed using Wilcoxon signed-rank tests. Changes in the EQ-5D-5L were analyzed using the paired t-test. Statistical significance was set at *p* ≤ 0.05 for all analyses (two-sided). Identified changes in outcomes between baseline and discharge were subsequently compared to established Minimal Clinically Significant Differences (MCID) for each outcome, where MCID had been previously described [39, 54–63]. Outcomes where MCID was achieved at discharge were noted.

The sample size was estimated pragmatically based on implementation at a single site over a short time frame, the facility’s current admission rate, the range of patients, the information required as to how long assessments would take, whether patients and staff could complete the chosen assessment tools and whether the tools identified changes in impairments in this population. This was designed to inform the choice of measurement tools for future conduction of a larger prospective study in the TCP population [64]. Whilst 6 to 8 participants were deemed feasible, the sample size was set at *n* = 12 to allow for loss to follow up or withdrawal, this yielded the data presented.

## Results

There were 23 admissions to restorative TCP at the facility during the recruitment period. Seventeen of the 23 older people approached met eligibility criteria and of those, five declined to participate. Twelve older people enrolled in the study but two withdrew prior to measurement due to unexpected discharge from their TCP. Therefore, ten older people (mean age 78.9 years, SD ± 9.1 years) completed baseline and discharge measurements (median LOS 46 days, IQR 26), further details of their characteristics are presented in Table 1. There were 10 staff who were eligible to participate in the study. Of those, five health professionals (one occupational therapist, two nurses, and two physiotherapists) enrolled in the study, with remaining staff citing lack of time available to participate due to additional commitments covering staff leave. Staff participants had a mean (standard deviation) of 13.4 (13.0) years of clinical practice experience and their mean length of experience working in transition care was 3 (2.4) years.

**Table 1:** Participant characteristics

Characteristics	Number of participants, *n* = 10 (100%)
Age 60–79 years	6 (60)
Age ≥ 80 years	4 (40)
Gender, male	6 (60)
Transition care length of stay (days), median (IQR)	46 (26)
Discharge destination	
Home	5 (50)
Residential aged care	5 (50)
Previous living situation	
Lived alone	6 (60)
Lived with partner	3 (30)
Lived with other people	1 (10)
Received pre-hospitalisation care support^a^	
ADL	1 (10)
IADL	8 (80)
Use of walking aids	
None	7 (70)
Wheeled walking frame	2 (20)
Wheelchair (non-ambulant)	1 (10)
Primary medical diagnosis	
Orthopaedic	3 (30)
Cardiorespiratory	2 (20)
Geriatric-related	2 (20)
Other	3 (30)
Mental health diagnosis (comorbidity)^b^	3 (30)
Falls history in last 12 months prior to admission to transition care	
No falls	3 (30)
1 fall	3 (30)
Multiple (≥ 2 falls)	4 (40)
Presence of visual impairments^c^	5 (50)

*SD* Standard deviation, *IQR* Interquartile range. All data reported as n (%) unless otherwise stated

^a^Pre-hospitalisation care support includes both formal and informal support

^b^Mental health diagnoses include bipolar disorder, depression and memory deficits

^c^Visual impairments include cataracts, glaucoma and macular degeneration

### Primary aim—evaluating the feasibility of using CGA to measure older peoples’ functional outcomes

The suite of assessments took 55.9 ± 7.3 min to complete. As the MoCA was administered separately, the time taken for completion was not included in combined data reported above. The occupational therapist estimated that between 30—60 min was required to complete the MoCA depending on the older adult’s cognitive ability.

All ten older people reported feeling positive regarding their overall assessment experience; specifically feeling capable of participating in the assessments and well informed regarding their current health status. They expressed satisfaction at knowing how they performed in the assessments. Most older people felt the assessment procedure was not too time consuming, however, one participant suggested that “30 min would be better” suggesting that a shorter duration might be a consideration. Six (60%) older people found the assessments easy to comprehend, while three (30%) commented that they lost a bit of focus due to the length and number of questions across all measures. One participant commented that some questions were “not usual questions that get asked.” Eight (80%) participants reported knowing their assessment findings helped them understand their own abilities and wellbeing better. For example, one participant stated, “it’s good to know what you’re capable of and if you’re improving.” Two older people also responded regarding participation in the assessments commenting, “it helped relieve boredom as there is nothing much to do at the facility”.

Details of the staffs’ evaluation regarding the suitability and acceptability of the selected suite of assessments are presented in Table 2. All staff (*n* = 5) reported that the assessment tools they administered were easy to complete, with zero adverse events reported during the study period. Staff agreed that the assessments provided useful information for rehabilitation and care planning. When administering the GDS staff perceived it was less acceptable for some older people, as questions regarding suicidal thoughts and death engendered some signs of discomfort.

**Table 2:** Evaluation of selected outcome measures for CGA

Domain	Outcome (*n* = 10 participants)	Number of participants assessed at admission/discharge (%)	Duration taken to complete measure (mins) (mean ± SD)	Evaluation comments from staff
Physical	10m Walk Test	9 (90%) / 9 (90%)	2.4 ± 0.53	- Minimal equipment required - Useful for quickly identifying gait impairments for further assessment and care planning - Walking speed score helpful for comparing with normative values for other functional correlates - Test condition of self-paced walking speed can be conducted relatively easily in patients with moderate to severe cognitive impairment as minimal instruction is required - One participant unable to perform 10MWT due to being non-ambulant (at least 3 years) prior to hospital admission
	Modified Barthel Index	100 (100%) / 100 (100%)	10.5 ± 1.54	- Completed by TC nurses at both admission and discharge - Provides useful information regarding personal ADL performance to assist with care planning
	de Morton Mobility Index	100 (100%) / 100 (100%)	14.3 ± 5.35	- Useful as part of initial and discharge assessment as it comprises of balance, bed mobility and ambulation measures - Useful as provides a comprehensive patient functional mobility profile for nursing and therapy staff management in a short amount of time - Hierarchy of tasks are useful in assisting to set smaller interim goals - Used across health and home care settings thus scores can be compared in longer term evaluation of patient functional mobility - Use will depend on baseline function pre-hospitalisation
	Timed Up and Go	9 (90%) / 9 (90%)	2.53 ± 0.96	- Easily completed at patient’s bedside - Provides very quick review of gait (walking), balance (turning) and leg strength (sit to stand) - Requires a patient to understand a 5-stage command hence low suitability for moderate cognitive impairment - One participant was unable to perform TUG due to being non-ambulant (at least 3 years) prior to hospital admission
Social	Lawton scale	100 (100%) / 100 (100%)	5.8 ± 2.17	- Provides useful information regarding older adults’ IADL performance to assist with planning for community discharge
	EQ-5D-5L	100 (100%) / 100 (100%)	3.6 ± 1.12	- Provides useful information regarding older adults’ self-perceived general health and wellbeing for program engagement
Cognitive	Mini Mental State Examination	100 (100%) / 100 (100%)	7.9 ± 2.88	- Easier to administer as it takes less time - Provide useful information on executive function, memory, orientation, language to facilitate communication - Has ceiling effect - Inclusion in assessment depends on type of client - Useful for older adults who are rarely assessed with MMSE
	Montreal Cognitive Assessment	100 (100%) / 100 (100%)	30–60	- Completed by TC occupational therapist - More sensitive in detecting mild cognitive impairment - Requires more time to assess
Emotional	Geriatric Depression Scale	100 (100%) / 100 (100%)	5.8 ± 3.14	- Can help screen patients for potential depressive symptoms at discharge that may require referral for services post-TCP discharge - Can make some patients feel slightly uncomfortable on specific questions - Questionnaire a bit long for administration - Less relevant for TCP clients; will not use as part of usual assessment unless indicated
	Patient Health Questionnaire-9	100 (100%) / 100 (100%)	6.4 ± 2.50	- Provides useful information for patients with potential depressive symptoms - While this measure focused on diagnostic criteria for DSM-IV depressive disorders, it is less repetitive and provoking - Assists clinicians to tailor activities for symptoms such as poor sleep, change in appetite and loneliness - Less relevant for these clients - Some questions appeared to make clients feel uncomfortable Less provoking and more general compared to GDS

### Secondary aim: evaluate if the assessments identified the impact of undertaking a facility-based TCP on older peoples’ functional outcomes across physical, mental, social and emotional domains

**Fig. 1:**
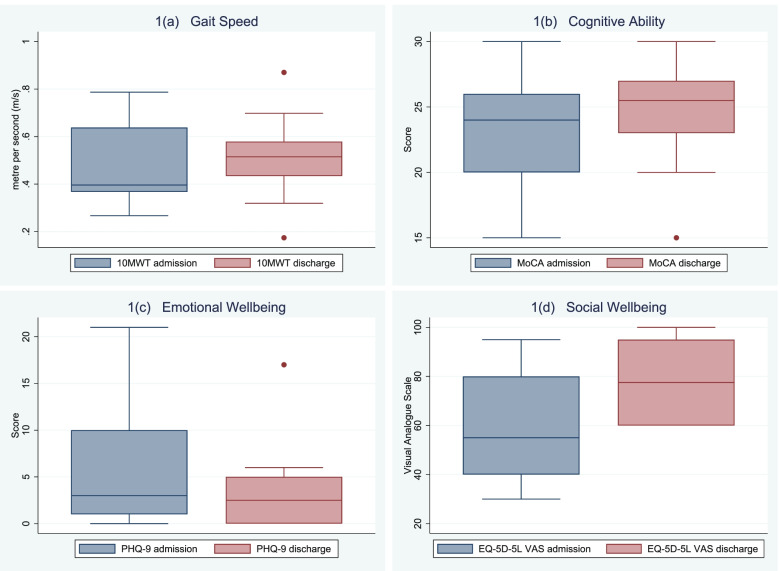
Changes in older adults’ functional outcomes measured at admission and discharge; **a** Gait speed, **b** Cognitive ability, **c** Emotional wellbeing, **d** Social wellbeing

Administration of the selected assessment tools showed they were able to identify changes in older peoples’ functional outcomes on undertaking facility-based TCP. Identified changes in older peoples’ functional outcomes are presented in Table 3 and identified changes in the 10MWT, EQ-5D-5L, MoCA and PHQ-9 assessments are also presented in Fig. 1. There were significant improvements at discharge compared to admission in: ADL performance measured by the MBI, gait speed measured by the 10MWT, mobility measured by the DEMMI and health-related quality of life as measured by the EQ-5D-5L. There was a significant reduction in depressive symptoms as measured by the GDS and a significant decline in IADL performance as measured by the Lawton scale at discharge compared to admission.

**Table 3:** Changes in older adults’ functional outcomes during a TCP

Domains	Outcome	Admission	Discharge	*p*-value
Physical	Modified Barthel Index^a^	71.50 (16.00)	76.50 (14.00)	0.008^c^
	de Morton Mobility Index^a^	41.50 (23.00)	55.00 (24.00)	0.011^c^
	Timed Up and Go^a^	25.16 s (23.37)^d^	21.88 s (15.47)^d^	0.859
	10m Walk Test^a^	0.40 m/s (0.27)^d^	0.52 m/s (0.15)^d^	0.008^c^
Social	Lawton scale^a^	6 (2)	4 (4)	0.013^c^
	EQ-5D-5L index value^b^ (mean ± SD)	0.63 ± 0.28	0.64 ± 0.27	0.751
	EQ-5D-5L health state score^b^ (mean ± SD)	59.00 ± 21.71	78.00 ± 16.02	0.007^c^
Emotional	Patient Health Questionnaire-9^a^ (mean ± SD)	5.60 ± 6.74	3.80 ± 5.16	0.100
	Geriatric Depression Scale^a^	4.00 (2.00)	1.50 (1.00)	0.011^c^
Cognitive	Mini Mental State Examination^a^	27.50 (1.00)	28.50 (5.00)	0.277
	Montreal Cognitive Assessment^a^	24.00 (6.00)	25.50 (4.00)	0.292

*SD* Standard deviation, *IQR* Interquartile range, *EQ-5D-5L* Five-level version of EuroQOL five-dimensional health-related quality of life. Data reported as median (IQR) unless otherwise stated

^a^Wilcoxon signed rank test used

^b^paired-t test used

^c^*p* < 0.05

^d^data collected for *n* = 9 participants only on admission and discharge, as one participant was non-ambulant

Mean changes for each functional outcome compared to established MCID are presented in Table 4. Older people demonstrated clinically significant improvements in two of four outcomes in the physical domain and one outcome in each of the social, cognitive and emotional domains. Conversely, there was a clinically significant decline in IADL performance as measured by the Lawton scale.

**Table 4:** Changes in the MCID for functional outcome measures during TCP

Domains	Outcome	MCID	Change observed
Physical	Modified Barthel Index	1.85 points [54]	5.0 points^a^
	de Morton Mobility Index	12.0 points [39]	14.5 points^a^
	Timed Up and Go	3.4s [55]	3.28 s
	10m Walk Test	0.14 m/s [56]	0.12 m/s
Social	Lawton scale	0.5 points [57]	-7.0 points^a^
	EQ-5D-5L index value	0.06–0.16 [58]	0.01
	EQ-5D-5L health state score	8–11 points [58]	19 points^a^
Emotional	Patient Health Questionnaire-9	5 points [59]	1.8 points
	Geriatric Depression Scale	2 points [60]	2.5 points^a^
Cognitive	Mini Mental State Examination	1.6–2.0 points [61]	1.0 point
	Montreal Cognitive Assessment	1.22–2.0 points [62, 63]	1.50 points^a^

*MCID* Minimal clinically significant difference, *EQ-5D-5L* Five-level version of EuroQOL five-dimensional health-related quality of life [39, 54–63],  = MCID reported in reference population

^a^Clinically significant change

## Discussion

Findings from this study demonstrated that it was feasible to measure older peoples’ functional outcomes using CGA in a residential transition care setting, enabling a holistic, tailored approach to rehabilitation programming. The use of CGA in different health settings, such as hospitals, and rehabilitation facilities with multidisciplinary teams has been shown to allow patients to receive holistic care from appropriate healthcare professionals [31, 32]. To our knowledge the feasibility of using CGA in TCP has not been previously reported, although recent reviews of TCP in Australia (including facility-based TCP) suggest a more comprehensive and holistic assessment of older people is required [12, 17, 29]. The outcome measures chosen for inclusion in CGA were able to identify clinically significant changes in older peoples’ function in the physical, social, cognitive and emotional domains at program completion. Our study employed an observational design, whereby the CGA was assessed for its feasibility to identify changes made by older participants and not to tailor treatment or alter TCP. However, the inclusion of CGA in settings other than TCP has been found to assist therapists tailor rehabilitation to meet the needs of community-dwelling older people, making programs more effective in reducing frailty and improving functional outcomes [50, 65].

### Acceptability and suitability of procedures and assessment measures

All assessments were reported as acceptable as they provided useful clinical information, were easily administered and caused no adverse events. Staff felt that overall, the assessments were suitable for older people undertaking a facility-based TCP with minimal floor or ceiling effects. The PHQ-9 was deemed a more suitable primary assessment of wellbeing compared with the GDS in this population, as it was perceived to be more conducive to older people for disclosing their emotional needs. Although assessment tools such as the TUG and 10MWT were useful in identifying underlying impairments and activity limitations causing poor physical function in ambulant individuals [44], some older people were unable to undertake them due to being non-ambulant on admission. Completing CGA was found to require an increased time commitment compared with the current assessment procedure. The research conditions also meant eight assessments were administered in a single contact session, which was a little overwhelming for some participants. However, the short duration taken to complete each individual assessment, when shared amongst the multidisciplinary staff, administered at different contact times could contribute to improved acceptability. While it was not always possible to match the most appropriate health professional with the CGA tool (e.g. clinical psychologist to conduct GDS) due to work roster availability at the time an older adult was recruited, this did not hinder the completion of CGA. Importantly, older people who completed CGA expressed satisfaction at knowing how they performed across the range of assessments. They commented that the assessments provided them with a better understanding of their health and wellbeing and the relevance of rehabilitation, making them feel more confident to participate in TCP. Older people in US inpatient settings similarly expressed the need to understand the reasons and potential benefits for undertaking further rehabilitation, rather than returning home, in order to commit to program participation [66]. Three participants expressed that they felt they lost some focus due the length of the questions, which could be due to fatigue related to medical illness, reduced health literacy or cognitive impairments. However, all were able to complete the assessments in a reasonable time frame.

### Changes in older peoples’ functional outcomes

The MBI, which is mandated in TCP, identifies older peoples’ limitations with ADL performance represented in the physical domain of function only [24]. The inclusion of outcome measures representing the other domains of function, as part of CGA, informs healthcare providers regarding an older adult’s social, cognitive and emotional wellbeing contributing to a more holistic picture of function [32]. The older people in our study recorded slower times on the 10MWT and TUG gait measures compared with normative values for community-dwelling older people [44]. Gait speeds are correlated with physical aspects of function, such as the ability to rise from a chair or safely cross a road [44]. The slower gait speed findings suggest that these measures could be useful for evaluation of older people in facility-based TCP to identify which individuals could benefit from additional rehabilitation aimed at improving mobility for safer and successful return to community living [43, 44]. The assessment tools chosen could also provide useful information about falls risk, which is known to be a major problem in this population [5–7]. Gait speed and the TUG test provide information about mobility, strength and balance impairments all of which are established falls risk factors among older people, especially those with a previous history of falls [51, 67]. Older peoples’ IADL performance, as measured by the Lawton scale, declined during the study period. A prior trial also reported that participation in hospital-based transitional rehabilitation had no impact on older peoples’ IADL performance [68]. This suggests completing an IADL assessment such as the Lawton scale could be important. If a gap exists, where a decline in IADL performance is not addressed by the current rehabilitation components making up a TCP, this could increase the risk of the older person having poorer functional outcomes, such as reduced independence or unplanned admissions to RAC [1–3]. A study in the Netherlands explored the implementation of short term residential care, which included admissions from hospital for further rehabilitation, with similar aims to facility-based TCP in Australia [18]. This study found that pre-existing functional decline is rarely addressed and that good treatment for this and other problems is required in intermediate care settings [18].

Clinically significant changes were observed in six of the ten selected outcome measures. Similar findings were reported in previous studies of TCP, where clinically significant changes were reported for gait speed and the functional mobility [10, 23, 39]. The ability of the selected outcome measures to detect MCIDs is also important as it means the TCP was able to elicit a clinical improvement that was meaningful and beneficial to the older adult [44].

### Strengths and limitations of the research

A key strength of this study was having facility staff conduct the suite of assessments, as their understanding of and experience in TCP enabled them to assess and evaluate whether they were suitable for the TCP population. The assessment tools chosen are frequently used by health professionals in a broad range of settings [42–47, 64–56]. However, due to research time constraints we were unable to access the discipline-specific health professional staff member to complete the relevant assessment at all times (e.g. Clinical psychologist conducting the GDS) meaning some assessments were completed by another health professional. We acknowledge that assessments were only conducted at admission and discharge, which was a pragmatic consideration to reflect the likelihood of CGA being implemented within current workloads, future work could consider measurement at additional timepoints. Another key strength of the study was that older people participated and provided a patient’s perspective about the feasibility of undertaking these assessments. Participants included older people with different primary diagnoses and mobility levels that reflected the characteristics of those admitted to the facility-based TCP [12].

Limitations of the study were the small sample size and single site. The small sample size for the study may have potentially contributed to inaccuracies in the changes detected at discharge from TCP, such as the decline in IADL performance. While the suite of assessments proved feasible to conduct in our facility-based setting, findings could be better generalized to populations undertaking TCP with a larger sample size, involving more facilities and settings across different countries. One participant was non ambulant and therefore could not complete two assessments (TUG and 10mwt). However, their other functional outcomes were able to be evaluated using all other measures, including HRQOL and self-care. Therefore, their data were included as it was felt that this older person was representative of a sub-cohort of the population admitted to TCP in Australia.

We did not include older people with moderate or severe dementia. However, these older adults form a smaller proportion of the admissions to residential transition care and may be admitted to await residential care placement, rather than undertake rehabilitation [12]. This is an area for future study as inclusion of cognitive assessments (MMSE and MoCA), which are validated and used in rehabilitation settings, can help identify patients with cognitive deficits that may benefit from therapy and hence tailor programs accordingly [29, 30]. There was a lack of established MCIDs based on TCP populations for most of the selected outcome measures. Hence the outcome measures were selected based on their use in previous TCP studies, as well as their validity, reliability and clinical suitability for the TCP population [10, 23, 39–41]. While it was feasible to use these assessment tools to measure changes in function, applying a CGA did not cause improvements in function in this cohort. In our study the CGA did not contribute to therapists’ decision making and was not used to guide treatment. Further research is required to determine if CGA could be used to tailor treatments in TCP and if this would result in improvement in function compared to not using CGA. A systematic review found that using CGA in home and hospital settings could lead to better health outcomes for older people [65].

## Conclusions

This study demonstrated that measuring older peoples’ functional outcomes using CGA was feasible in a transition care setting. Staff found the outcome measures provided useful information and that the selected assessments were able to preliminarily identify clinically significant changes across physical, social and emotional domains. These findings may be useful for health professionals to develop a suite of assessments that assist them in tailoring rehabilitation for older peoples’ functional needs prior to discharge, facilitating a smoother transition to community living. Future research with a larger sample size, across a range of TCP settings and countries is required to evaluate the impact of CGA in TCP on improving functional outcomes for older people.

## Supplementary Information

**Additional file 1: Table 1.** Summary of assessment tools used as part of comprehensive geriatric assessment.

## Data Availability

The datasets used and/or analyzed during the current study cannot be made publicly available as participants did not provide consent for their data to be shared. Further details regarding the data and ethical conditions for access are available from the corresponding author on reasonable request.
