# Supplementary material for: Using comprehensive geriatric assessment for older adults undertaking a facility-based transition care program to evaluate functional outcomes: a feasibility study
Source: BMC Geriatr. 2022 Jul 19;22:598. doi: 10.1186/s12877-022-03255-5 (PMC9294817; doi:10.1186/s12877-022-03255-5)
Supplement: Supplementary file 1 — Additional file 1: Table 1. Summary of assessment tools used as part of comprehensive geriatric assessment. [file 12877_2022_3255_MOESM1_ESM.docx]

**eTable 1.** Summary of assessment tools used as part of comprehensive geriatric assessment

| Domain | Component | Measurement or Assessment Tool | Description |
| --- | --- | --- | --- |
| Medical | Medications | Number of medications | Polypharmacy is associated with frailty, unplanned hospitalisations and mortality.^52^ |
|  | Diagnoses | Number and type of medical conditions | Presence of multiple medical conditions is associated with functional decline and poor ADL performance. |
| Physical | Independence of ADLs | Modified Barthel Index | 10 items scored out of 5, 10 or 15 depending on the task, and a total score out of 100 will be given. A higher score indicates greater independence. |
|  | Mobility | de Morton Mobility Index | 15 items scored from 0-2 to derive a raw score of out of 19, which will be converted to a transformed score of out of 100. Community dwelling older adults score 82 on average. A higher score indicates greater functional ability. |
|  | Functional Ability | Timed Up and Go | Time taken in seconds (s) to complete task. Normal range = 8-11s, lesser time taken indicates better functional ability. |
|  | Gait speed | 10-metre Walk Test | Time taken to walk 10m as fast and safe as possible. A shorter timing indicates faster gait speed and better performance. |
|  | Falls | Falls history for previous 12 months | History of falls associated with poor outcomes such as decreased function, morbidity and mortality.^34^ |

| Social | Living Situation | Discharge destination | Home, residential aged care or hospital. |
| --- | --- | --- | --- |
|  | Instrumental Activities of Daily Living (IADL) | Lawton scale | 8 domains with scored 0 or 1, with a total score of 8. Individuals scored according to their highest level of function in each domain. |
|  | Health-related quality of life (HRQoL) | EQ-5D-5L | Health states scored using 5 dimensions with 5 response levels for each dimension which is presented as a 5-digit index value. Self-rated health status measured using a visual analogue scale and scored from 0-100. |
| Emotional | Depression | Patient Health Questionnaire-9 | 9 questions scored from 0-3 and a total score out of 27 will be given. The higher the score, the greater the severity of depression. |
|  |  | Geriatric Depression Scale | 30 yes/no questions with a score of 1 given for each response that matches the provided answer at the end of the question. Score ranges from 1-15, score > 4 indicates presence of depressive symptoms. |
| Cognitive | Cognitive Ability | Mini Mental State Examination | Two sections assessing different domains of cognitive function, with a total score of 30. A lower score indicates greater cognitive impairment. |
|  |  | Montreal Cognitive Assessment | 12 tasks assessing different domains of cognitive function with a total score of 30. A lower score indicates greater cognitive impairment. |
